# Supplementary material for: Tackling Structural Complexity in Li2S-P2S5 Solid-State Electrolytes Using Machine Learning Potentials
Source: Nanomaterials (Basel). 2022 Aug 26;12(17):2950. doi: 10.3390/nano12172950 (PMC9458117; doi:10.3390/nano12172950)
Supplement: Supplementary file 1 [file nanomaterials-12-02950-s001.zip › nanomaterials-1836721-supplementary.pdf]

Supplementary Materials: Tackling Structural Complexity in Li_2S-P_2S_5 Solid-State Electrolytes using Machine Learning Potentials

Carsten G. Staacke [1,†], Tabea Huss [1,†], Johannes T. Margraf [1], Karsten Reuter [1] and Christoph Scheurer [1,2,*]

1. GAP Model Details

The GAP models used herein decompose the total energy into local two-body (2B) and many-body (MB) contributions.[1] The MB contributions are based on the Smooth Overlap of Atomic Positions (SOAP) kernel, which provides a rotationally and permutationally invariant measure of the similarity of chemical environments.[2] Technical hyperparameters used for the two-body and SOAP descriptors are listed in table S1.

Description	Symbol	2B	SOAP
Cutoff	r_{cut} [Å]	5	6
Transition Width	Δ [Å]	0.6	0.7
Atom Smearing	σ_{at} [Å]	-	0.7
SOAP Basis Size	n_{max}/l_{max}	-	4/12
Kernel Exponent	ζ	-	2
Size of Sparse Set	N_{sparse}	20	2000

Table S1. Technical Hyperparameters for the GAP models used in this work.

2. Data Efficient Training Protocol

The employed training protocol is taken from previous work and likewise employed for Li_3PS_4 and $Li_7P_3S_{11}$.[3] This procedure is based on a Voronoi tessellation of the anion grid, yielding high symmetry positions as potential Li sites. An exemplary tessellation is shown in Fig. S1. Using k-means clustering, diverse set of these sites can be generated. The underlying assumption of this procedure is a relative rigidity of the thiophosphates and high mobility of Li ions. This assumption is not in contrast to anion lattice dynamics, such as the paddlewheel effect.[4] Although, small anions increase the conductivity by quasi continuous rotations, thiophosphates only exhibit no translational motion.[4]

This potential is then used to generate new configurations via molecular dynamics (MD) simulations at 800 K, which are added to the training set. This procedure is repeated for several iterations (termed "generations") until the force and energy errors on new configurations no longer improve. The GAP model is extend by the β Li_3PS_4 modification using the same iterative training approach. α and γ crystal configurations are used as test cases.

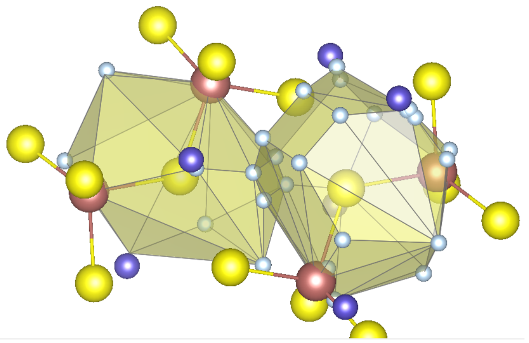

Figure S1. Voronoi Tesselation of the anion grid. Li (darkblue), P (red), S (orange) and Voronoi vertices(light blue). Figure is readapted from [3]

To build amorphous counterparts for the crystalline structures, a Monte Carlo-like sampling approach is chosen. This approach allows for the free tuning of stoichiometries and anions. Only the dominant anions (e.g. PS_4^{3-} and $P2S_7^{4-}$) are taken into consideration in the first place and utilized in a ratio that represents correctly the desired stoichiometry. The random sampling approach is depicted Fig. S2. Thiophosphate anions are rotated by a random angle and placed randomly on a predefined grid. Alternatively we used a Poisson disc sampling for a homogeneous placement of anion, yielding similar results as the placement on a grid. Thereupon, lithium ions are added into the structure utilizing a Voronoi tesselation of the sulfur anion grid and chosen to ensure charge neutrality accordingly. The hereby obtained cell is then compressed to yield desired experimental densities range. This is accomplished by iterative rescaling of the cell by a factor in all three directions in alternation with a subsequent geometry optimizations utilizing the GAP model. To consequently obtain a glassy structure that represents the physical system, short molecular dynamics runs of the glasses at a temperature of 600 K - 800 K are conducted and the iterative training proceeded on these geometries, similarly to the training of crystalline geometries.

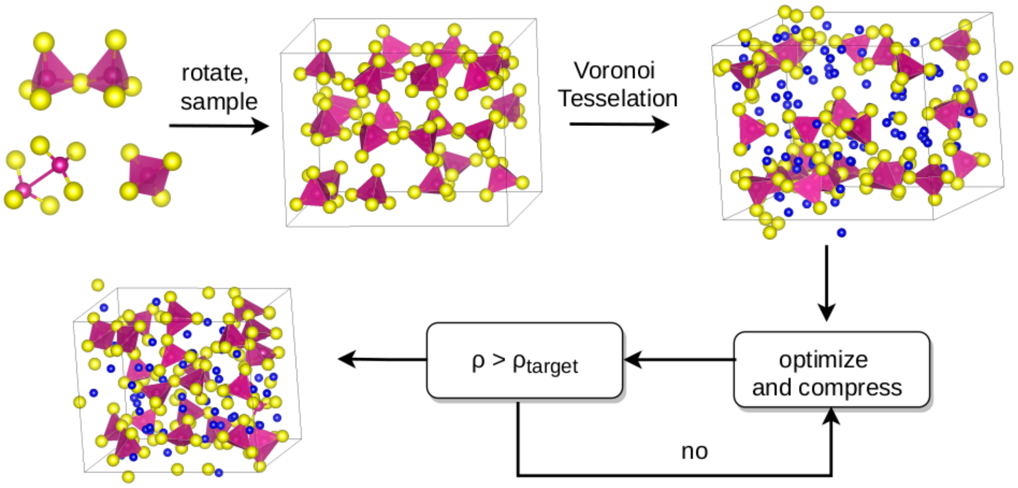

Figure S2. Graphical representation of the sampling approach, involving random sampling of thiophosphate compounds on a predefined grid, addition of lithium ions via a Voronoi Tesselation of the anion grid, and iterative compressing to the desired density. Lithium is displayed in blue, phosphorous in violet, and sulfur in yellow.

In a final step the GAP model is extended by additional local P-S microchemistry. In particular, the emphasis is set on the $P_2S_6^{4-}$ anion which has been found frequently especially in glassy materials.

The extension of by P_2S_6 is necessary as we observe the $P_2S_6^{4-}$ units to degrade during the optimization when isolated S atoms are added to the training but tend to either form structures which resemble the $P_2S_7^{4-}$ anion or collapse the P-P bond to distances below 0.5 Å. This can be intuitively explained as the GAP model had not been trained on different P-P bonds before and can hence not predict the energy profile on the P-P coordinate correctly. To prevent this unphysical behavior, the P-P bond distance is systematically sampled by distorting the $Li_4P_2S_6$ crystal and training the GAP on these geometries. It is indeed found that the untrained GAP minimizes P-P distances. The minimized P-P distance is in contrast to the expected coulombic behavior in DFT. When retraining on P-P data, however, the GAP correctly preidtcs the P-P binding energy. The distorted structures and the corresponding energy profiles of the $Li_4P_2S_6$ crystal, untrained and trained GAP are provided in Figure S3 Glasses are sampled in a similiar fashion as above by using PS_4^{3-} and $P_2S_6^{4-}$ anions in a ratio of 1:1 and adding Li ions accordingly.

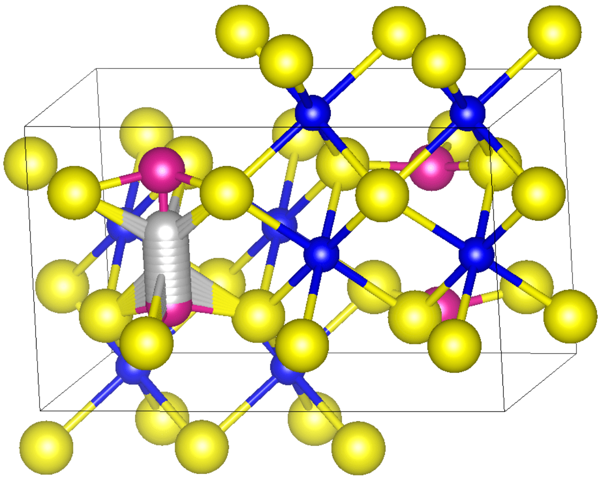

Figure S3. Systematic sampling of P-P distances in the $Li_4P_2S_6$ crystal. Geometries used for the sampling approach: The initial structure is distorted by displacing one phosphorous atom (i.e. shortening the P-P bond length). Lithium is displayed in blue, phosphorous in purple, sulfur in yellow, and the phosphorous atoms in the distorted structures in light grey.

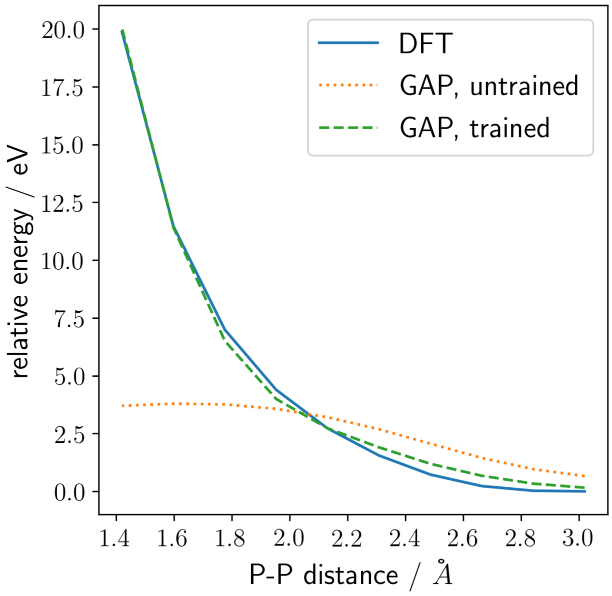

Figure S4. Obtained energy profile from DFT, untrained, and trained GAP as a function of P-P distance.

3. Convergence and Error Assessment

Convergence is reached at RMSEs of 0.3 eV/ and 7.5 meV/atom for forces and energies. The energy and force RMSEs obtained for the sampled amorphous structures are significantly higher than the values obtained for the equilibrated ones. This may be explained by the high (local) anisotropy of the training configurations: lithium ions are not evenly distributed and coordinated by a sphere of sulfur atoms, but often occur in clusters. The higher anisotropy is expected to increase the locality error induced by the finite cutoff of the SOAP descriptor.[**?**] To consequently obtain a glassy structure that represents the physical system, short molecular dynamics runs of the glasses at a temperature of 600 K - 800 K are conducted. Force and energy convergence in the iterative training cycle is shown in Figure S2. A significant decrease in force and energy RMSEs is observed as converged RMSEs of 0.2 eV/ and 5 meV/atom are predicted.

The peak in RMSE energy of the validation set in generation 5 can be explained by the different temperature chosen for the MD run in this generation, which was 1000 K instead of 600 K. An elevated temperature, upon which the GAP is not yet trained, leads to a systematic underestimation of the energy. This effect is to be expected also for MD production runs at different temperatures. When correcting/retraining the GAP predicted energy for the observed offset, one obtains an energy RMSE of 5 meV/atom.

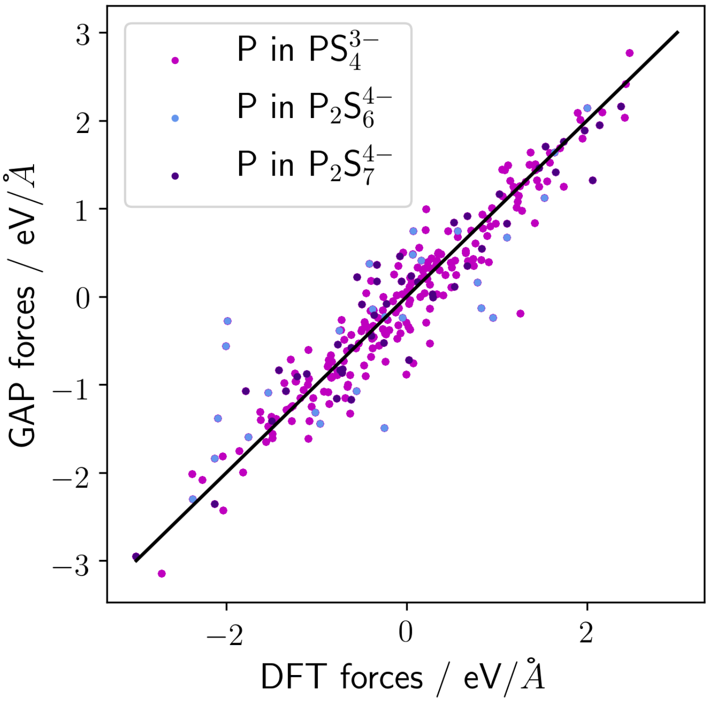

Figure S5. Coordination-resolved force correlation plot for P atoms in MD snapshots of $Li_7P_3S_{11}$ glasses.

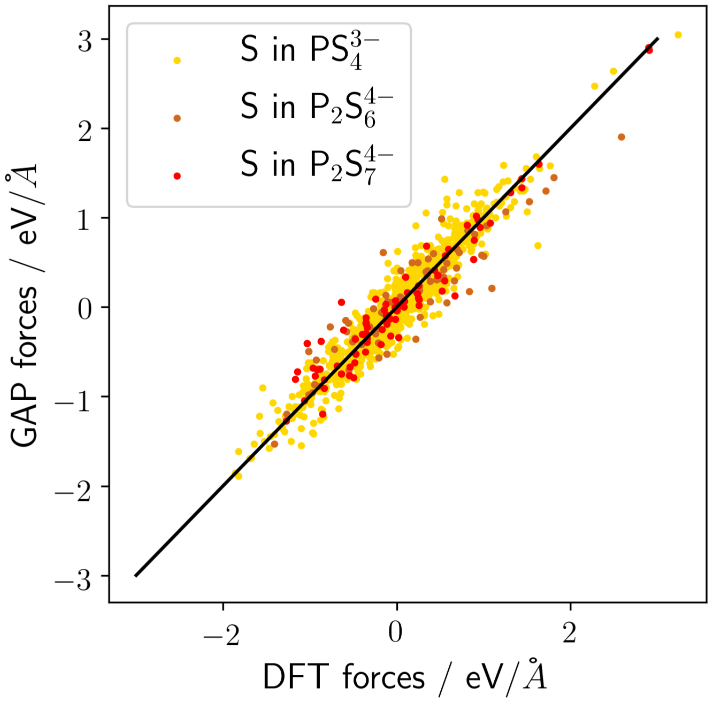

Figure S6. Coordination-resolved force correlation plot for S atoms in MD snapshots of $Li_7P_3S_{11}$ glasses.

A comparison of force RMSEs of phosphorous and sulfur in different P_mS_n polyhedra (Figure S6 and Table S2) proves, that RMSEs in $P_2S_6^{4-}$ are elevated in comparison to those in PS_4^{3-} and $_2S_7^{4-}$. This may be attributed to the different charge states of phosphorous and sulfur or to non-ideal hyperparameters for this polyhedron.

P_mS_n	RMSE P / eV/Å	RMSE S / eV/Å
PS_4^{3-}	0.36	0.24
$P_2S_6^{4-}$	0.67	0.31
$P_2S_7^{4-}$	0.38	0.18

Table S2. Coordination-resolved force RMSEs of phosphorous and sulphur in MD snapshots of $Li_7P_3S_{11}$ glasses.

4. Radial Distribution Functions

The element-resolved radial distribution functions for glassy Li_3PS_4 along with the crystalline counterparts (α-, β-, and γ-Li_3PS_4) are displayed in Figure **??**.

It is apparent that the P-S and S-Li radial distribution functions of glass and crystal are identical. Differences occur for S-S, where a double peak at around 4 Åis observed for the β- and γ-crystal, but neither for the α-phase nor for the amorphous structures. Kim *et al.* have used this peak in literature to discriminate between hexagonal and cubic S-sublattices in the structure.[5] The P-P RDF displays different structural feature: In the amorphous material two peaks are observed which are not found in the crystals, located at 2 - 2.5 Åand a broadening of the distinct crystalline peaks at 4.5 - 6.5 Å. These features are attributed to the occurrence of $P_2S_6^{4-}$ and $P_2S_7^{4-}$ moieties.

Another distinction can be made for the peaks at $r_{\text{P-P}} \leq 4.5$ Å: Two distinct peaks occur in all crystal phases, while one broad peak is visible for the glass. This observation serve as a validation for the non-periodicity of the P-lattice in the amorphous material.

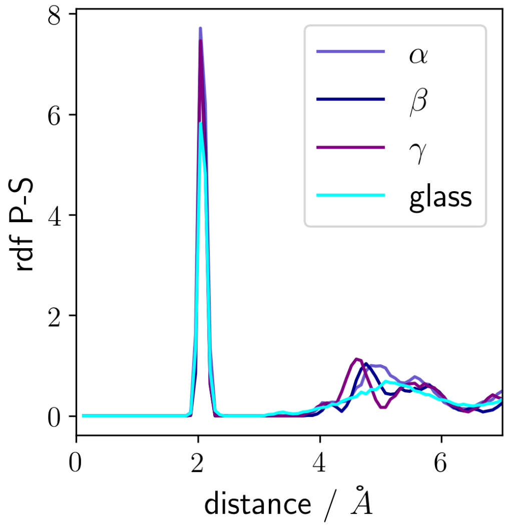

Figure S7. Radial distribution functions for a) P-S, collected from molecular dynamics runs of α, β, and γ-crystals at 500K, using an averaged ensemble of 20 different Li_3PS_4 glasses with ρ = 1.8 g/cm^3.

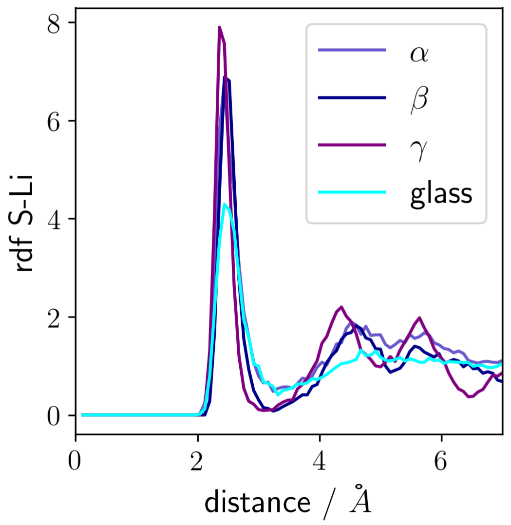

Figure S8. Radial distribution functions for b) S-Li.

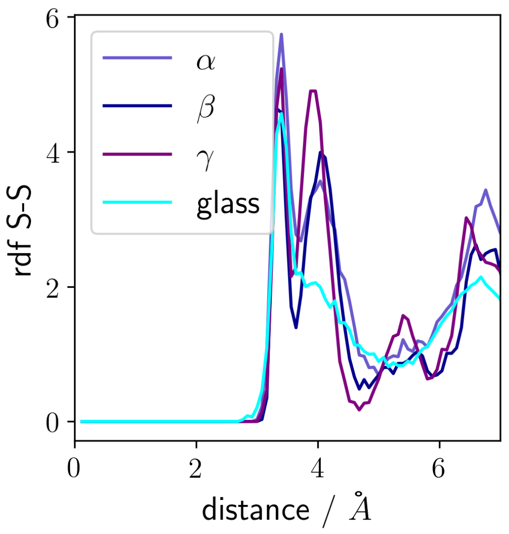

Figure S9. Radial distribution functions for c) S-S.

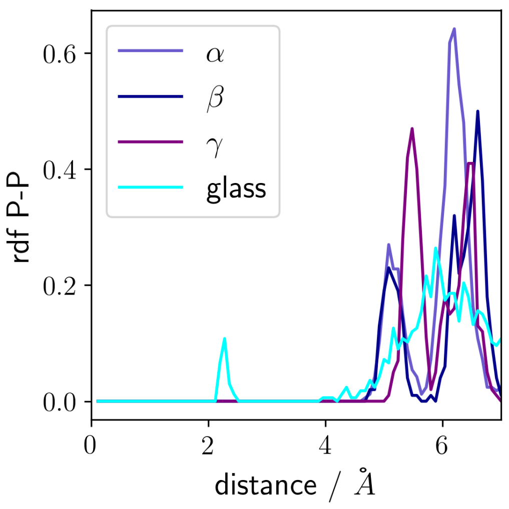

Figure S10. Radial distribution functions for d) P-P.

5. Li_3PS_4

Li_3PS_4 is the crystalline compound of Li_2S = 75 mol% and can be found in three different phases: α, β, and γ -Li_3PS_4. All of them solely contain the simplest PS_4^{3-} anion.

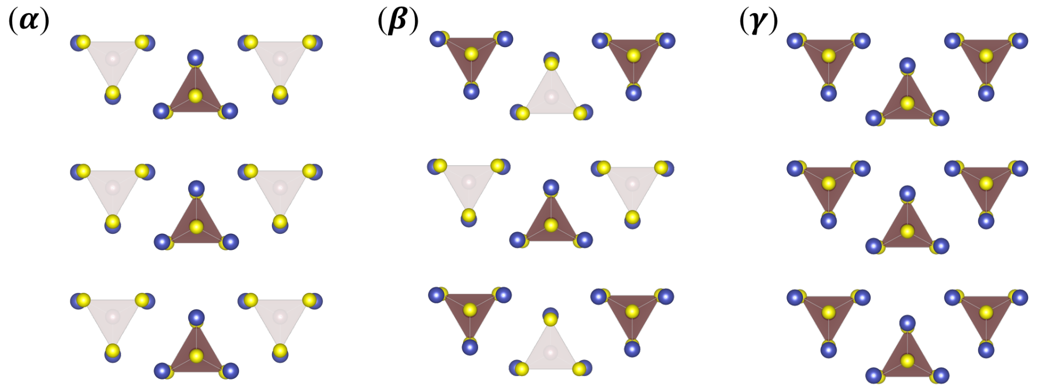

Figure S11. Arrangements of PS_4 tetrahedra in the α, β, and γ in Li_3PS_4. (Li: blue; S: yellow; P: red)

6. Ionic Mobility

As a measure for the ion transport, we evaluate the tracer diffusion coefficients D^*_α from the Einstein relation as

$$D^*_\alpha = \frac{1}{3}\frac{\langle |r_i(t) - r_i(0)|^2 \rangle}{2\Delta t} \quad (1)$$

where $r_i(t)$ is the position of particle i at time t and Δt is the sampling time.[6] In addition to D^*, the ionic conductivity σ^* is determined via the Nernst-Einstein formulation based on the tracer diffusion coefficients as

$$\sigma^* = \frac{1}{3Vk_BT}\sum_{\alpha}^{n_\alpha} q_\alpha^2 N_\alpha D^*_\alpha \quad (2)$$

where V is the volume, T equals temperature, n_α the number of different species α, q_α the charge, N_α the number of ions, and D^*_α the tracer diffusion coefficients.[7]

For LPS glasses a range of densities have been reported. At first we exemplary evaluate the lithium ion conductivity as a function of the materials density at 600K. As shown in Figure S12 we can't identify a clear trend. The weak trend is not pronounced in comparison to the width of the distribution.

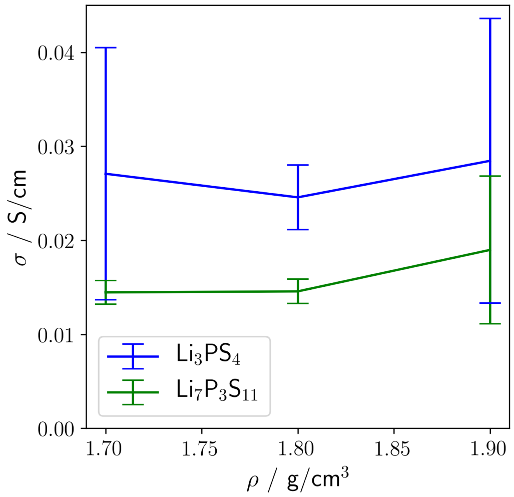

Figure S12. Density dependence of the conductivity of Li_3PS_4 and $Li_7P_3S_{11}$ glasses, using an ensemble of three structures for each density and stoichiometry at 600K.

A measure for the amorphous nature of the glasses is isotropicity with respect to the ion diffusivity in all dimensions. This is tested using an ensemble of 20 MD runs of Li_3PS_4 glass at 500 K. It is found that the standard deviation of the mean square displacement in x, y, and z direction vary by 9 % in one MD run, but by only 1 % when averaging over the 20 structures in the ensemble. Hence, the structures are sufficiently isotropic with respect to diffusivity.

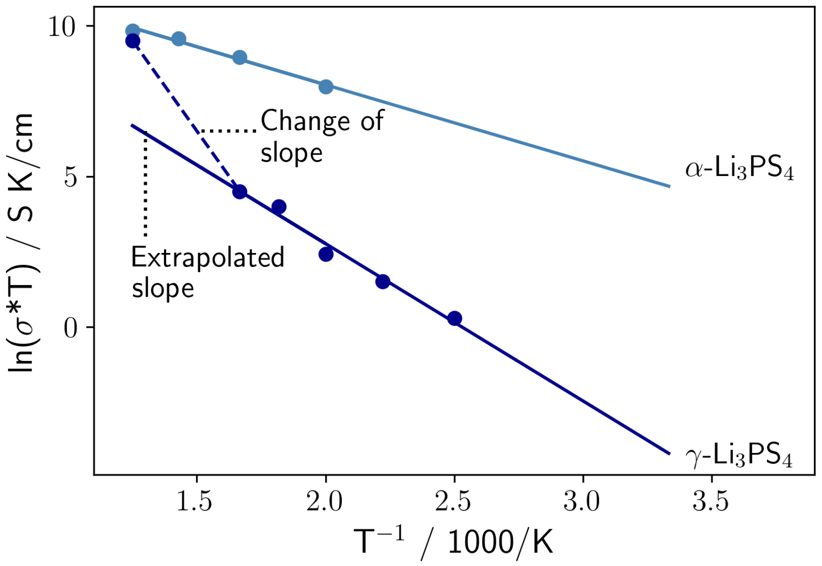

Figure S13. Computational Arrhenius plots for α (light blue solid line) and γ (dark blue line) phase of Li_3PS_4. γ-Li_3PS_4 exhibits a change of slope at roughly 700 K. Above 700 K, conductivities of γ-Li_3PS_4 matches those of α-Li_3PS_4.

Table S3. Summarized conductivities from MD calculations performed in this study of the ternary LPS system. The abbreviations *AIMD*, *MD* and *Exp* denote the reference methods. The top part corresponds to crystalline LPS, bottom glassy LPS. A detailed list of synthesis methods and ionic conducitivities can be found in Ref. [8].

phase	σ_{RT} / S/cm	σ_{RT}, lit. / S/cm	E_A / eV	E_A, lit. / eV
$Li_7P_3S_{11}$	$2.9 \pm 1.1 \times 10^{-3}$	5.6×10^{-2}, *AIMD* [9]<br>1.2×10^{-2}, *Exp* [9]<br>1.7×10^{-2}, *Exp* [10]	0.21 ± 0.01	0.19, *AIMD* [9]<br>0.21, *Exp* [9]<br>0.17, *Exp* [10]
α-Li_3PS_4	$3.6 \pm 2.9 \times 10^{-3}$	0.8×10^{-2}, *AIMD* [5]	0.22 ± 0.02	0.18, *AIMD* [5]
β-Li_3PS_4	$6.0 \pm 0.1 \times 10^{-5}$	8.9×10^{-7}, *Exp* [11]<br>10^{-1}, *AIMD* [12]	0.48 ± 0.04	0.46,*Exp* [13]<br>0.16, *Exp* [11]<br>0.08, *AIMD* [12]<br>0.40, *AIMD* [14]
γ-Li_3PS_4	$5.5 \pm 0.4 \times 10^{-7}$	3.0×10^{-7}, *Exp* [11][13]	0.42 ± 0.16	0.49, *Exp* [13]<br>0.22, *Exp* [11]
Li_3PS_4	$7.4 \pm 0.9 \times 10^{-4}$	4.1×10^{-4}, *MD* [15]<br>8.8×10^{-5}, *AIMD* [16]<br>2.8×10^{-4}, *Exp* [17]<br>7.5×10^{-4}, *Exp* [18]	0.280	0.28, *MD* [15]<br>0.17, *AIMD* [16]<br>0.40, *Exp* [17]
$Li_7P_3S_{11}$	$4.1 \pm 0.8 \times 10^{-4}$	8.2×10^{-5}, *AIMD* [16]<br>3.7×10^{-5}, *Exp* [17]<br>1×10^{-4}, *Exp* [19]	0.267	0.45, *Exp* [17]
$Li_4P_2S_7$	$2.4 \pm 0.2 \times 10^{-4}$	5.7×10^{-5}, *AIMD* [16]<br>3.8×10^{-5}, *Exp* [17]	0.278	0.44, *Exp* [17]

7. Li accessible volume

In the main script we analyzed the number of Li-positions occupied during MD simulations at finite temperatures by calculating the isosurface of the probability density distribution of Li-positions. In Figure S14 we visualize the accessible volume. Exemplary we choose the glasses with the highest Li content (Li_3PS_4) and the lowest Li content ($Li_4P_2S_7$). Li_3PS_4 has a higher accessible Li-volume at all temperatures compared to $Li_4P_2S_7$.

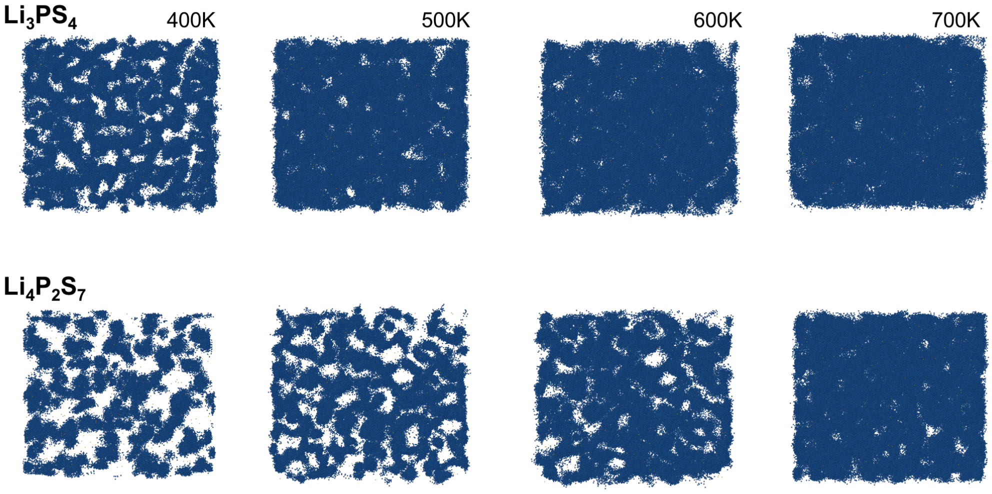

Figure S14. Li accessible volume (blue) for $Li_4P_2S_7$ and Li_3PS_4 at given temperatures.

1. Bartók, A.P.; Payne, M.C.; Kondor, R.; Csányi, G. Gaussian approximation potentials: The accuracy of quantum mechanics, without the electrons. *Phys. Rev. Lett.* **2010**, *104*, 136403. https://doi.org/10.1103/PhysRevLett.104.136403.
2. Bartók, A.P.; Kondor, R.; Csányi, G. On representing chemical environments. *Phys. Rev. B* **2013**, *87*, 184115. https://doi.org/10.1103/PhysRevB.87.184115.
3. Staacke, C.G.; Heenen, H.H.; Scheurer, C.; Csányi, G.; Reuter, K.; Margraf, J.T. On the Role of Long-Range Electrostatics in Machine-Learned Interatomic Potentials for Complex Battery Materials. *ACS Appl. Energy Mater.* **2021**, *4*, 12562–12569. https://doi.org/10.1021/acsaem.1c02363.
4. Smith, J.G.; Siegel, D.J. Low-temperature paddlewheel effect in glassy solid electrolytes. *Nat. Comm.* **2020**, *11*, 1–11. https://doi.org/10.1038/s41467-020-15245-5.
5. Kim, J.S.; Jung, W.D.; Choi, S.; Son, J.W.; Kim, B.K.; Lee, J.H.; Kim, H. Thermally Induced S-Sublattice Transition of Li_3PS_4 for Fast Lithium-Ion Conduction. *J. Phys. Chem. Lett.* **2018**, *9*, 5592–5597. https://doi.org/10.1021/acs.jpclett.8b01989.
6. Mehrer, H. *Diffusion in solids: fundamentals, methods, materials, diffusion-controlled processes*; Vol. 155, Springer Science & Business Media, 2007.
7. Frenkel, D.; Smit, B. *Understanding molecular simulation: from algorithms to applications*; Vol. 1, Elsevier, 2001.
8. Kudu, Ö.U.; Famprikis, T.; Fleutot, B.; Braida, M.D.; Le Mercier, T.; Islam, M.S.; Masquelier, C. A review of structural properties and synthesis methods of solid electrolyte materials in the Li_2S- P_2S_5 binary system. *J. Power Sources* **2018**, *407*, 31–43. https://doi.org/10.1016/j.jpowsour.2018.10.037.
9. Chu, I.H.; Nguyen, H.; Hy, S.; Lin, Y.C.; Wang, Z.; Xu, Z.; Deng, Z.; Meng, Y.S.; Ong, S.P. Insights into the performance limits of the $Li_7P_3S_{11}$ superionic conductor: a combined first-principles and experimental study. *ACS Appl. Mater. Interfaces* **2016**, *8*, 7843–7853. https://doi.org/10.1021/acsami.6b00833.
10. Seino, Y.; Ota, T.; Takada, K.; Hayashi, A.; Tatsumisago, M. A sulphide lithium super ion conductor is superior to liquid ion conductors for use in rechargeable batteries. *Energy Environ. Sci.* **2014**, *7*, 627–631. https://doi.org/10.1039/c3ee41655k.
11. Homma, K.; Yonemura, M.; Kobayashi, T.; Nagao, M.; Hirayama, M.; Kanno, R. Crystal structure and phase transitions of the lithium ionic conductor Li_3PS_4. *Solid State Ion.* **2011**, *182*, 53–58. https://doi.org/10.1016/j.ssi.2010.10.001.
12. Yang, Y.; Wu, Q.; Cui, Y.; Chen, Y.; Shi, S.; Wang, R.Z.; Yan, H. Elastic Properties, Defect Thermodynamics, Electrochemical Window, Phase Stability, and Li^+ Mobility of Li_3PS_4: Insights from First-Principles Calculations. *ACS Appl. Mater. Interfaces* **2016**, *8*, 25229–25242. https://doi.org/10.1021/acsami.6b06754.
13. Tachez, M.; Malugain, J.; Mercire, R.; Robert, G. Ionic conductivity of and phase transition in lithium thiophosphate Li_3PS_4. *Solid State Ion.* **1984**, *14*, 181–185. https://doi.org/10.1016/0167-2738(84)90097-3.

14. de Klerk, N.J.; van der Maas, E.; Wagemaker, M. Analysis of Diffusion in Solid-State Electrolytes through MD Simulations, Improvement of the Li-Ion Conductivity in β-Li_3PS_4 as an Example. *ACS Appl. Energy Mater.* **2018**, *1*, 3230–3242. https://doi.org/10.1021/acsaem.8b00457.
15. Kim, J.S.; Jung, W.D.; Son, J.W.; Lee, J.H.; Kim, B.K.; Chung, K.Y.; Jung, H.G.; Kim, H. Atomistic assessments of lithium-ion conduction behavior in glass–ceramic lithium thiophosphates. *ACS Appl. Mater. Interfaces* **2018**, *11*, 13–18. https://doi.org/10.1021/acsami.8b17524.
16. Baba, T.; Kawamura, Y. Structure and ionic conductivity of Li_2S–P_2S_5 glass electrolytes simulated with first-principles molecular dynamics. *Front. Energy Res.* **2016**, *4*, 1–22. https://doi.org/10.3389/fenrg.2016.00022.
17. Dietrich, C.; Weber, D.A.; Sedlmaier, S.J.; Indris, S.; Culver, S.P.; Walter, D.; Janek, J.; Zeier, W.G. Lithium ion conductivity in Li_2S-P_2S_5 glasses – building units and local structure evolution during the crystallization of superionic conductors Li_3PS_4, $Li_7P_3S_{11}$ and $Li_4P_2S_7$. *J. Mater. Chem.A* **2017**, *5*, 18111–18119. https://doi.org/10.1039/c7ta06067j.
18. Shiotani, S.; Ohara, K.; Tsukasaki, H.; Mori, S.; Kanno, R. Pair distribution function analysis of sulfide glassy electrolytes for all-solid-state batteries: Understanding the improvement of ionic conductivity under annealing condition. *Sci. Rep.* **2017**, *7*, 1. https://doi.org/10.1038/s41598-017-07086-y.
19. Seino, Y.; Nakagawa, M.; Senga, M.; Higuchi, H.; Takada, K.; Sasaki, T. Analysis of the structure and degree of crystallisation of 70Li2S–30P2S5 glass ceramic. *J. Mater. Chem. A* **2015**, *3*, 2756–2761. https://doi.org/10.1039/c4ta04332d.
